# Fast Object Tracking on a Many-Core Neural Network Chip

**DOI:** 10.3389/fnins.2018.00841

**Published:** 2018-11-16

**Authors:** Lei Deng, Zhe Zou, Xin Ma, Ling Liang, Guanrui Wang, Xing Hu, Liu Liu, Jing Pei, Guoqi Li, Yuan Xie

**Affiliations:** ^1^Department of Precision Instrument, Center for Brain Inspired Computing Research, Tsinghua University, Beijing, China; ^2^Department of Electrical and Computer Engineering, University of California, Santa Barbara, Santa Barbara, CA, United States

**Keywords:** object tracking, many-core architecture, neural network chip, recurrent neural networks, attractor dynamics

## Abstract

Fast object tracking on embedded devices is of great importance for applications such as autonomous driving, unmanned aerial vehicle, and intelligent monitoring. Whereas, most of previous general solutions failed to reach this goal due to the facts that (i) high computational complexity and heterogeneous operation steps in the tracking models and (ii) parallelism-limited and bloated hardware platforms (e.g., CPU/GPU). Although previously proposed devices leverage neural dynamics and near-data processing for efficient tracking, their flexibility is limited due to the tight integration with vision sensor and the effectiveness on various video datasets is yet to be fully demonstrated. On the other side, recently the many-core architecture with massive parallelism and optimized memory locality is being widely applied to improve the performance for flexibly executing neural networks. This motivates us to adapt and map an object tracking model based on attractor neural networks with continuous and smooth attractor dynamics onto neural network chips for fast tracking. In order to make the model hardware friendly, we add local-connection restriction. We analyze the tracking accuracy and observe that the model achieves comparable results on typical video datasets. Then, we design a many-core neural network architecture with several computation and transformation operations to support the model. Moreover, by discretizing the continuous dynamics to the corresponding discrete counterpart, designing a slicing scheme for efficient topology mapping, and introducing a constant-restricted scaling chain rule for data quantization, we build a complete mapping framework to implement the tracking model on the many-core architecture. We fabricate a many-core neural network chip to evaluate the real execution performance. Results show that a single chip is able to accommodate the whole tracking model, and a fast tracking speed of nearly 800 FPS (frames per second) can be achieved. This work enables high-speed object tracking on embedded devices which normally have limited resources and energy.

## 1. Introduction

Object tracking is important for many applications including autonomous driving, unmanned aerial vehicle, intelligent monitoring, etc. The object tracking models used by prior work can be clustered into several categories: discriminative or generative models (Li et al., 2013; Wang N. et al., 2015), machine learning models (Grabner et al., 2008; Wang and Yeung, 2013; Hare et al., 2016), and dynamic neural models (Faubel and Schöner, 2008; Spencer and Perone, 2008; Wu et al., 2008; Martel and Sandamirskaya, 2016). The generative models leverage specific characteristics to represent the object, i.e., using representative methods such as the PCA (Ross et al., 2008; Wang et al., 2013) and sparse coding methods (Jia et al., 2012; Zhang T. et al., 2012), while the discriminative models separate the object from the backgrounds by training binary classifier (Kalal et al., 2012; Zhang K. et al., 2012). To improve the tracking accuracy, various machine learning algorithms, such as boosting (Grabner et al., 2008), structured output SVM (Hare et al., 2016), and correlation filter (Bolme et al., 2010; Henriques et al., 2015) have been applied. Recently, deep learning, convolutional neural network in particular, has shown the ability to automatically extract high-level features and improve the accuracy significantly (Wang and Yeung, 2013; Hong et al., 2015; Held D. et al., 2016; Wang et al., 2017). However, these emerging neural network (NN) algorithms are usually very demanding in terms of compute and memory resources, limiting their execution speed. In addition, many of these algorithms usually involve several separate steps with heterogeneous operations to construct a complete tracking model (Gurcan and Temizel, 2015; Wang et al., 2017), which affects the hardware compatibility of all these different operations. To realize fast object tracking still remains as a challenge but important for applications such as motion posture capture in sports field (Chen et al., 2015; Pueo, 2016), cell imaging and movement analysis in biomedical field (Beier and Ibey, 2014), and some real-life scenarios (Galoogahi et al., 2017a). Compared to above complex models, the recurrent neural networks (RNNs) with attractor dynamics (Faubel and Schöner, 2008; Spencer and Perone, 2008; Wu et al., 2008; Martel and Sandamirskaya, 2016) are more promising for fast tracking. They are capable of holding a continuous family of stationary states and form a continuous manifold wherein the dynamic behavior is neutrally stable, facilitating the smoothness of the object tracking. The compact and end-to-end paradigm promises efficient hardware implementation.

Another factor limiting the tracking speed comes from the hardware aspect. It is well known that conventional CPU and GPU platforms suffer from von Neumann bottleneck limited by the memory bandwidth. Furthermore, these platforms are usually bloated to keep the programming flexibility for general purpose applications. These characteristics together with their bulky size and huge energy consumption make it difficult for the embedded deployment. Previous work (Martel and Sandamirskaya, 2016) implemented neural dynamics on dedicated vision chip for efficient tracking benefit from the near-data processing. Whereas, the flexibility of programming and application is limited due to the tight integration with the vision sensor and the effectiveness on various video datasets is yet to be fully demonstrated. Recently, many-core architecture for efficient execution of NN models has been widely demonstrated (Merolla et al., 2014; Shi et al., 2015; Chi et al., 2016; Shafiee et al., 2016). Via parallel computation and optimized memory locality, many-core architectures can achieve high throughput and power efficiency. Besides, the support for various neural network structures and inter-chip communication brings better flexibility and potential scalability, respectively. This motivates us to adapt and map an end-to-end NN model onto a many-core chip for fast object tracking.

However, we should note that a many-core NN architecture usually suffers from some hardware constraints, such as limited connections and data precision, which must be addressed prior to model deployment. To this end, first, we adapt an RNNs-based object tracking model to make it hardware-friendly. Then, we design a many-core NN architecture with five vector/matrix operations and three transformation operations to support the model computation. In order to deploy the tracking model, we propose several optimization techniques: (1) to address the fan-in and fan-out limitation of the single core, we add a local connection restriction that makes the model more hardware-friendly and use a slicing scheme for efficient topology mapping; (2) to implement the differential equations in digital circuits, we discretize the continuous temporal dynamics to the corresponding discrete counterpart; (3) to meet the requirement of fixed-point data with limited bit width, we propose a constant-restricted scaling chain rule for model quantization. Comprehensive evaluations of the model accuracy on various tracking datasets are demonstrated, and a real chip is fabricated for validation. Results show that a fast tracking speed of nearly 800 FPS (frames per second) can be achieved. The compact size and high efficiency present great potential for intelligence on embedded devices, especially in the scenarios that require high-speed object tracking.

The rest of the paper is organized as follows. Section 2 provides backgrounds for the tracking model and the hardware-friendly modification. Section 3 presents the design of many-core NN architecture. How to deploy the tracking model onto hardware is illustrated in section 4. Then, comprehensive evaluations on the tracking accuracy and system performance are conducted in section 5. Finally, this work is concluded and discussed in section 6.

## 2. Hardware-friendly tracking model

In this section, we provide backgrounds for the tracking model we use in this paper and introduce local-connection restriction. We select an RNN model proposed by Wu et al. (Fung et al., 2008, 2010; Wu et al., 2008) named continuous attractor neural network (CANN), which is a neuroscience-inspired model. In fact, similar models with self-sustaining neural dynamics, termed as dynamic neural fields (DNF), can also be found in Faubel and Schöner (2008), Spencer and Perone (2008), Martel and Sandamirskaya (2016) and Schöner and Spencer (2016) where the only difference is the format of inhibition function.

**Figure 1:**
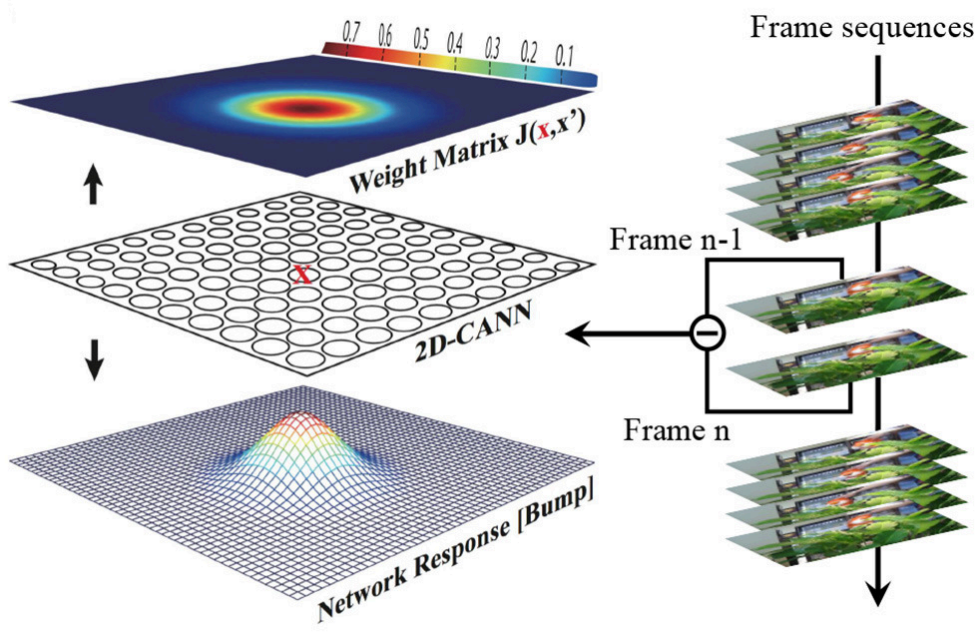
Illustration of the CANN model.

We first review the original dynamic model of a two-dimensional (2D) CANN, as shown in Figure 1. Denote **x** as a coordinate position on the 2D plane, *V*(**x**, *t*) as the membrane potential of the neuron at position **x** and time *t*, and *r*(**x**, *t*) as the firing rate of this neuron. It is reasonable to assume that *r*(**x**, *t*) increases along with *V*(**x**, *t*), but saturates in the presence of global inhibition. A model that captures this feature obeys

(1) $$r\left(x,t\right)=\frac{V^{2}\left(x,t\right)}{1+k\int_{-\infty }^{+\infty }V^{2}\left(x^{\prime },t\right)dx^{\prime }}$$

where *k* is a small positive hyper-parameter that controls the strength of global inhibition.

Let *V*_*ext*_(**x**, *t*) be the external stimulus to neuron **x** at time *t*. In the CANN model, *V*(**x**, *t*) is determined by the external stimulus and the recurrent inputs from other neurons, and its own relaxation, which is governed by

(2) $$\tau \frac{\partial V\left(x,t\right)}{\partial t}=-V\left(x,t\right)+\beta \int_{-\infty }^{+\infty }J\left(x,x^{\prime }\right)r(x^{\prime },t)dx^{\prime }+V_{ext}\left(x,t\right)$$

where τ is a time constant, which is typically at the magnitude of 1 ms, and β determines the ratio between the recurrent inputs and the external stimulus. *J*(**x**,**x**′) is the neuronal interaction (synaptic weight) from the neuron at location **x**′ to the neuron at location **x**. *J*(**x**,**x**′) is configured as

(3) $$J\left(x,x^{\prime }\right)=\frac{J_{0}}{2\pi a^{2}}e^{-\frac{|x-x^{\prime }{|}^{2}}{2a^{2}}}$$

where *J*_0_ is a constant, *a* denotes the Gaussian interaction range, |**x** − **x**′| represents the Euclidean distance between neuron **x** and **x**′. $\frac{J_{0}}{2\pi a^{2}}$ is the maximum interaction. We can see that equation (3) encodes a synapse pattern (bump shape) with translational invariance, producing a similar response bump pattern represented by large fire rates of neurons. The response bump implies where the object is. Furthermore, the neuronal distance is circular, which means that the most top and bottom neurons, as well as the most left and right neurons, are connected as adjacent neurons. This symmetry guarantees the bump stability at the boundary.

**Figure 2:**
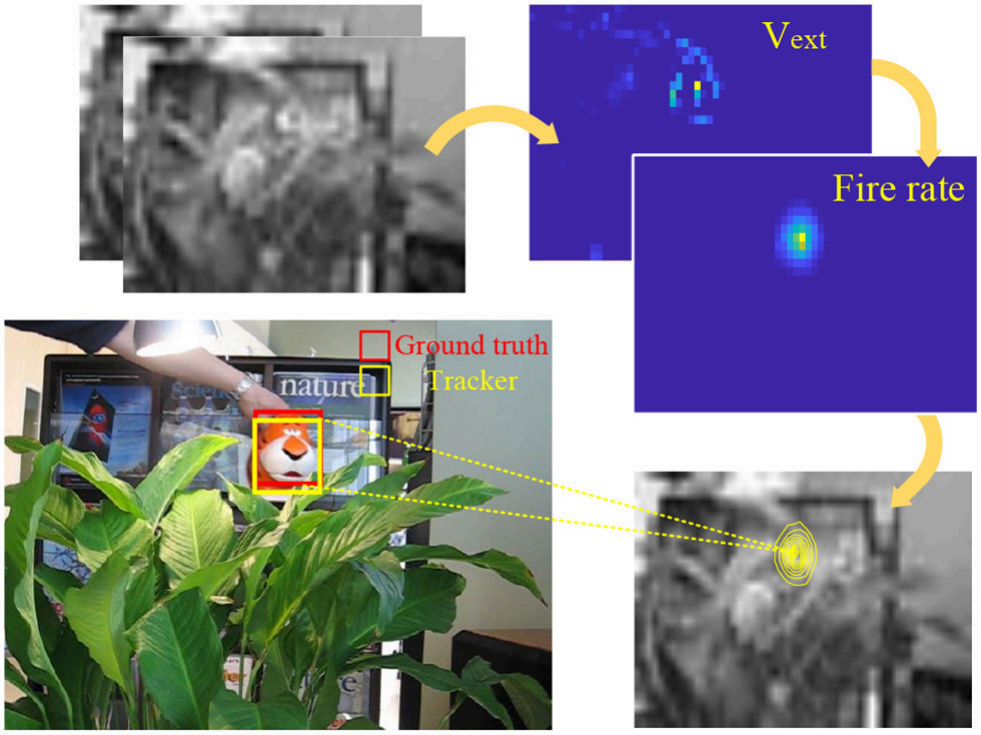
Illustration of CANN-based object tracking.

The overview of CANN model is shown in Figure 1, where the bump-shape pattern of synaptic connections and fire rates forms a hallmark feature. The difference signal of every two adjacent frames from the video is injected into the network as the external stimulus in Equation (2). Each neuron receives the intensity of the corresponding pixel in the 2D difference frame. CANN model is able to track objects smoothly because of the continuous neural dynamics that results in a smooth moving trajectory of the response bump. The trajectory presents as: (1) in the absence of external stimulus, the network can still keep a fixed response bump via recurrent injection; (2) in the presence of an object, especially a continuously moving one, the network can smoothly shift its response bump in accordance with the moving target. Here the external stimulus acts as the object to be tracked, and the neuronal response bump indicates the predicted object location. Figure 2 illustrates the tracking process. The red bounding box is the ground truth of the object location, and the yellow bounding box represented by the response bump reflects the predicted location. The original high-resolution video is resized to the CANN network scale before running the tracking model.

However, the above CANN model is not hardware friendly when we map it onto a hardware for real-time tracking. One of the major obstacles is the huge connections in the model. Take a network with 1000 neurons as an example, if they are fully connected, there are one million connections causing a huge wiring overhead. In this section, we introduce a distance-aware local connection to address this issue. Actually, for practical hardware implementation, other constraints are also required to be solved, such as mapping differential equation onto digital circuit, changing floating point operation to fixed point one with limited data bit width, partitioning the whole computational graph to sub-graphs for mapping it onto the many-core architecture, which will be explained in latter sections.

### 2.1. Distance-aware local connection

Despite the interconnection limitation from hardware, each neuron has strong connections only within the Gaussian bump field (usually a circle) as shown in Equation (3). In this sense, the remote connections usually have small impact on the neuronal membrane potential and fire rate. Therefore, it is possible to remove the remote connections without much accuracy loss. To this end, we propose a distance-aware local connection topology, as below

(4) $$J\left(x,x^{\prime }\right)=\{\begin{array}{ll} \frac{J_{0}}{2\pi a^{2}}\cdot e^{-\frac{|x-x^{\prime }{|}^{2}}{2a^{2}}}, & \text{if neuron}\;x^{\prime }\in CF\left(x,R\right)\\ 0, & \text{otherwise} \end{array}$$

where **x**′ ∈ *CF*(**x**, *R*) represents that each neuron **x** is only locally connected to its neighboring neurons within a *R* × *R* rectangle area centered by **x**. We term this local area as connection field (CF). The local pruning modification reduces many remote connections to save interconnection resources in the following chip implementation. The rectangle shape rather than circle is for matching with the slicing scheme for efficient mapping that will be introduced in section 4.2.

## 3. Many-core neural network architecture

**Figure 3:**
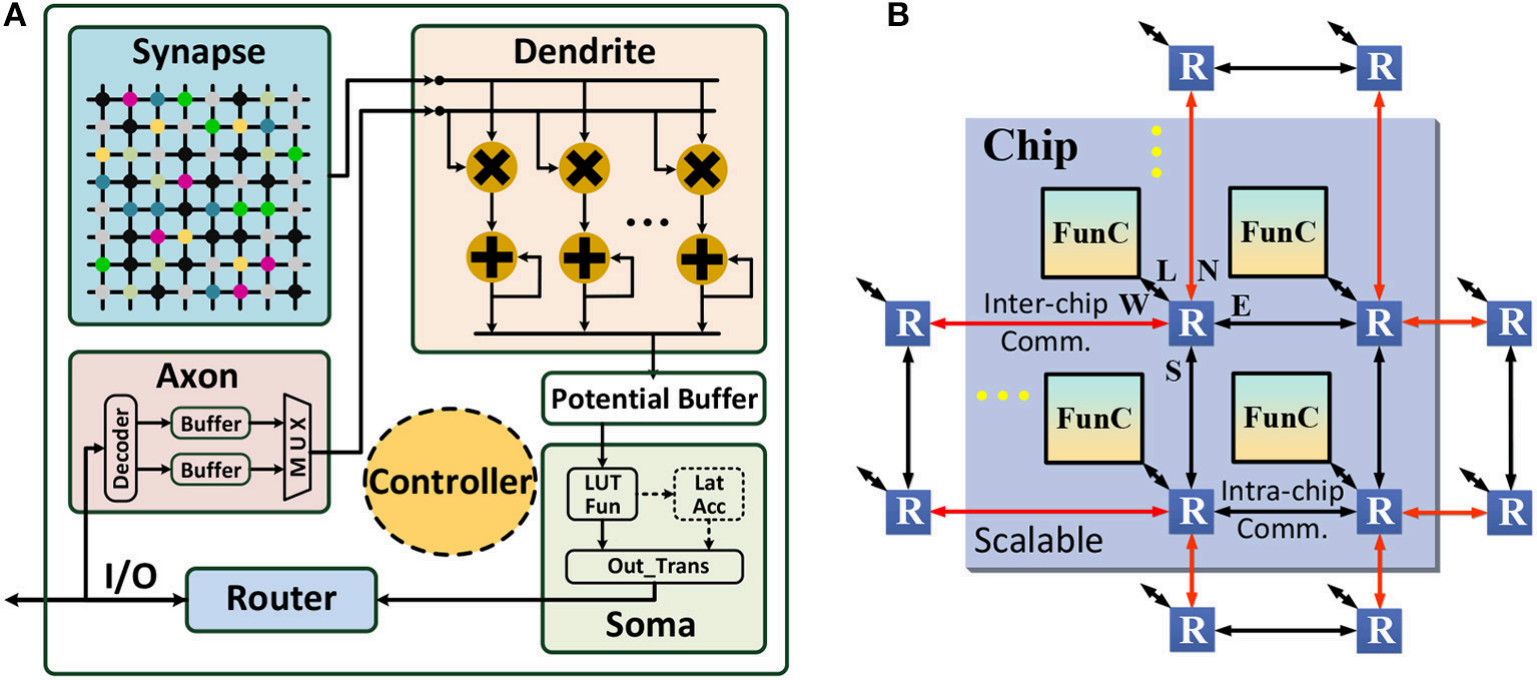
Many-core neural network architecture: **(A)** functional core (FunC); **(B)** scalable many-core network.

As aforementioned, the many-core NN architecture holds great potential for high throughput because of the extreme processing parallelism with decentralized cores and improved memory locality without off-chip memory access. Usually, this architecture consists two levels of design: (1) functional core (FunC) that is a small self-contained NN for supporting various vector/matrix arithmetic operations; (2) many-core network wired by a scalable routing infrastructure. Here we design a many-core NN architecture shown in Figure 3 for implementing the CANN-based object tracking on chip.

### 3.1. Functional core

The basic computation in NNs are the vector/matrix operations, such as vector-matrix multiplication (VMM) or vector-vector addition/multiplication, which should be well supported by the basic building block in an NN architecture. Therefore, the FunC in this paper can be viewed as a compute engine for vector/matrix arithmetic operations. The emerging non-volatile memory (NVM) devices (Yu, 2018) have demonstrated great potential to build ideal FunC with efficient processing of the mentioned vector/matrix operations, via integrating both computation and memory on the same memory crossbar in analog domain (Chi et al., 2016; Shafiee et al., 2016; Ambrogio et al., 2018). However, the large-scale fabrication of this “physical crossbar” is still challenging. Therefore, we use a fully digital design with memory array and additional processing elements (e.g., multipliers and accumulators) to simulate the crossbar-like dataflow, which can be treated as “virtual crossbar". The fully digital design is able to save fabrication cost and reduce development period. Despite of this development simplification, our mapping framework for implementing CANN-based tracking is suitable for any many-core NN architecture, no matter what device technology is used.

Next, we introduce our architecture design. As shown in Figure 3A, each FunC is comprised of six units, including axon, synapse, dendrite, soma, router, and controller. Specifically, axon acts as a data buffer and provides the input for dendrite, as well as buffers the output from router (generated by soma). It has two SRAM chunks (256 × 8b for each), that act as two ping-pong buffers switching between the router write and the dendrite read. Synapse locally stores the connection weights (256 × 256 × 8b), which is logically organized as a crossbar and physically placed near to the dendrite computing for memory locality optimization. Dendrite is an integration engine occupying 16 8-bit multipliers and 16 24-bit accumulators, and soma is another computing block for neuronal transformation. Besides intra-FunC computing and data movement, inter-FunC communication is wired by routers. The overall dataflow follows: “remote FunC or local FunC ⇒ router ⇒ axon/synapse ⇒ dendrite ⇒ soma ⇒ router ⇒ local FunC or remote FunC”, and the controller manages the execution state machine.

As shown in Table 1, we design five operations in dendrite, including VMM (vector-matrix multiplication), VVM (vector-vector multiplication), VVA (vector-vector accumulation), VS (vector scaling), and VB (vector buffer), and three transformations in soma, including LUT_Fun (look up table function), Lat_Acc (lateral accumulation), and Out_Trans (output transmission). Thus, it is able to cover all the arithmetic requirements in the CANN model. In particular, for the 256 columns in the synapse array, the calculation is divided into 16 groups (16 columns for each group). The column-wise execution within each group is parallel while the inter-group execution is serial. In VMM operation, at each cycle, dendrite reads one input from axon, reads 16 weights from 16 columns on the same row from the synapse array, and then concurrently executes 16 MACs (multiply and accumulate) that share the same axon data. In VVM operation, dendrite ignores axon and reads dynamic data (e.g., membrane potential) rather than static weight from synapse, and executes variable-variable multiplications. Considering the practical requirement of CANN model, VVM only supports two-vector multiplication. VVA bypasses the multipliers, and it supports up to 128-vector addition operation for dimensional reduction. VS operation only requires one input from axon (scaling factor) and one row of dynamic data from synapse. Synapse is totally disabled in VB operation, and dendrite only copies data from axon, which is usually used for timing alignment via data delay. Note that, for the element-wise vector operations (e.g., VVM, VVA and VS), the synapse array is split into two chunks (128 × 256 × 8b for each), which alternately holds dynamic inputs from router and provides input for the consequent dendrite computation, i.e., working as two ping-pong buffers like that in axon.

**Table 1:** Dendrite and soma operations.

**Unit**	**Operation**	**Definition**
	VMM	**y** = **W**·**x**
	VVM	**y** = **x_1_**⊙**x_2_**
Dendrite	VVA	$y=\sum_{i}x_{i},i=0,1,...,127$
	VS	**y** = *x*_α_**x**
	VB	**y** = **x**
	LUT_Fun	**y** = φ(**x**)
Soma	Lat_Acc	*y*_*j*_ = *x*_*j*_ + *y*_*j* − 1_
	Out_Trans	Send output to router

### 3.2. Scalable many-core network

FunC is a small self-contained NN with 256 neurons and 256 × 256 programmable synaptic connections. Larger NNs can be constructed by wiring multiple FunCs together through routers, as shown in Figure 3B. In this way, the hierarchical scalability, i.e., FunC⇒chip⇒board⇒system, is easily to be obtained. Specifically, a typical routing topology of 2D mesh, XY Point-to-point (P2P) routing (Merolla et al., 2014; Akopyan et al., 2015), is used. The communication in X direction has a higher priority than the Y direction. Each router has five channels: Local, East, West, North, and South. A routing packet starts from the source neuron to the destination neurons through two stages: (1) move to a target memory cell in intra- or inter-chip FunC; (2) fan out to the target neurons when the computation starts in that FunC (VMM and VS operation). The input sharing mechanism saves long-distance communication to a great extent, and the routing table in each router is reconfigurable to support arbitrary network topologies. A synchronous clock is required within each FunC, while asynchronous communication with handshaking is enough for inter-FunC communication. A global phase synchronization for a complete round of computation and communication is used for ensuring the correct timing schedule. Besides the P2P routing, we will introduce a multicast routing scheme in section 4.2.

## 4. CANN deployment

To deploy the modified CANN model onto the many-core NN architecture, we propose a mapping framework including dynamics discretization, topology mapping, and data quantization, which will be introduced in this section.

### 4.1. Discretization of the continuous dynamics

Since digital circuits cannot directly support the continuous differential dynamics in Equation (2), we propose an iterative state update method for discretizing the continuous dynamics to an equivalent difference equation so that we can implement it in an iterative manner. By setting τ = 1 and ∂*t* = 1, the continuous state update of CANN can be modified to an iterative version of

(5) $$\{\begin{array}{l} V(x,t+1)=\beta \sum_{x^{\prime }\in CF}J(x,x^{\prime })\cdot r(x^{\prime },t)+V_{ext}(x,t)\\ r(x,t+1)=\frac{V^{2}(x,t+1)}{k\sum_{x^{\prime }}V^{2}(x^{\prime },t+1)} \end{array}.$$

Note that we always constrain the membrane potential to be positive, i.e., *V*(**x**, *t*) ≥ 0, and we change the term $1+k\underset{{\text{x}}^{\prime }}{\sum }V^{2}\left({\text{x}}^{\prime },t+1\right)$ to $k\underset{{\text{x}}^{\prime }}{\sum }V^{2}\left({\text{x}}^{\prime },t+1\right)$ for simplification. *V*(**x**, *t*) ≥ 0 can be simply implemented through designing *ReLU* function of *ReLU*(*x*) = *max*(0, *x*) in LUT_Fun.

**Figure 4:**
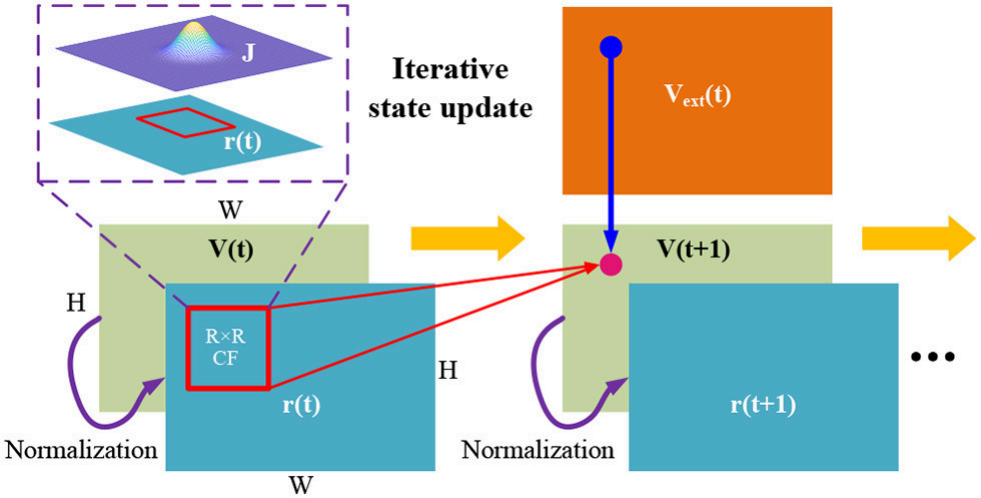
State update described by “{*r*(**x**, *t*) &*V*_*ext*_(**x**, *t*)} ⇒ *V*(**x**, *t* + 1) ⇒ *r*(**x**, *t* + 1) ⇒ … ” according to the discretized difference Equation (5).

Figure 4 presents the iterative state update of the above difference equation. The overall computational dataflow therefore becomes “{*r*(**x**, *t*) & *V*_*ext*_(**x**, *t*)} ⇒ *V*(**x**, *t* + 1) ⇒ *r*(**x**, *t* + 1) ⇒ x…”. Via above discretization, CANN model becomes realizable in digital circuit through the iterative execution.

### 4.2. Mapping of the network topology

To better understand the process of mapping the CANN topology onto the many-core NN architecture, we decompose each iteration of the difference Equation (5) into five steps as below.

**Five execution steps for each iteration**

1. Step 1 - recurrent input: $V_{1}\left(\text{x},t+1\right)=\beta \underset{{\text{x}}^{\prime }\in CF}{\sum }J\left(\text{x},{\text{x}}^{\prime }\right)\cdot r\left({\text{x}}^{\prime },t\right)$.
2. Step 2 - membrane potential: *V*(**x**, *t* + 1) = *ReLU*(*V*_1_(**x**, *t* + 1) + *V*_*ext*_(**x**, *t*)).
3. Step 3 - potential squared: *V*^2^(**x**, *t* + 1) = *V*(**x**, *t* + 1) · *V*(**x**, *t* + 1)
4. Step 4 - inhibition factor: $s_{inh}(t+1)=\frac{1}{k\sum_{x^{\prime }}V^{2}(x^{\prime },t+1)}$, and delay *V*^2^(**x**, *t* + 1).
5. Step 5 - firing rate: $r(x,t+1)=V^{2}(x,t+1)\cdot s_{inh}(t+1)$.

Among the five steps at each iteration, Step 1 consumes the most resources because of the expensive matrix multiplication while the other four steps only execute vector computation needing less resources. Therefore, here we provide the mapping details of this step and then briefly introduce the overall mapping scheme. Take a relatively small network as an example that includes 30 × 56 neurons where each one connects to all of its neighboring neurons in a 15 × 15 CF area. In Step 1, the neuronal outputs at time phase *t* will be fetched back to these neurons as inputs at next phase, and then participate in the generation of the next neuronal outputs at *t* + 1. The 30 × 56 inputs *r*(**x**′, *t*) and (30 × 56) × (15 × 15) synaptic weights *J*(**x**,**x**′) form a heavy VMM operation for achieving $V_{1}\left(\text{x},t\right)=\beta \underset{{\text{x}}^{\prime }}{\sum }J\left(\text{x},{\text{x}}^{\prime }\right)\cdot r\left({\text{x}}^{\prime },t\right)$. However, each FunC has a connection constraint with only 256 fan-ins and 256 fan-outs (determined by the size of synapse array), which makes it impossible to execute the large VMM on a single core. To reduce the resource requirements, we propose a slicing scheme for efficient topology mapping. Combined with the aforementioned distance-aware local connection, the slicing scheme further helps obtain a regular placement pattern.

**Figure 5:**
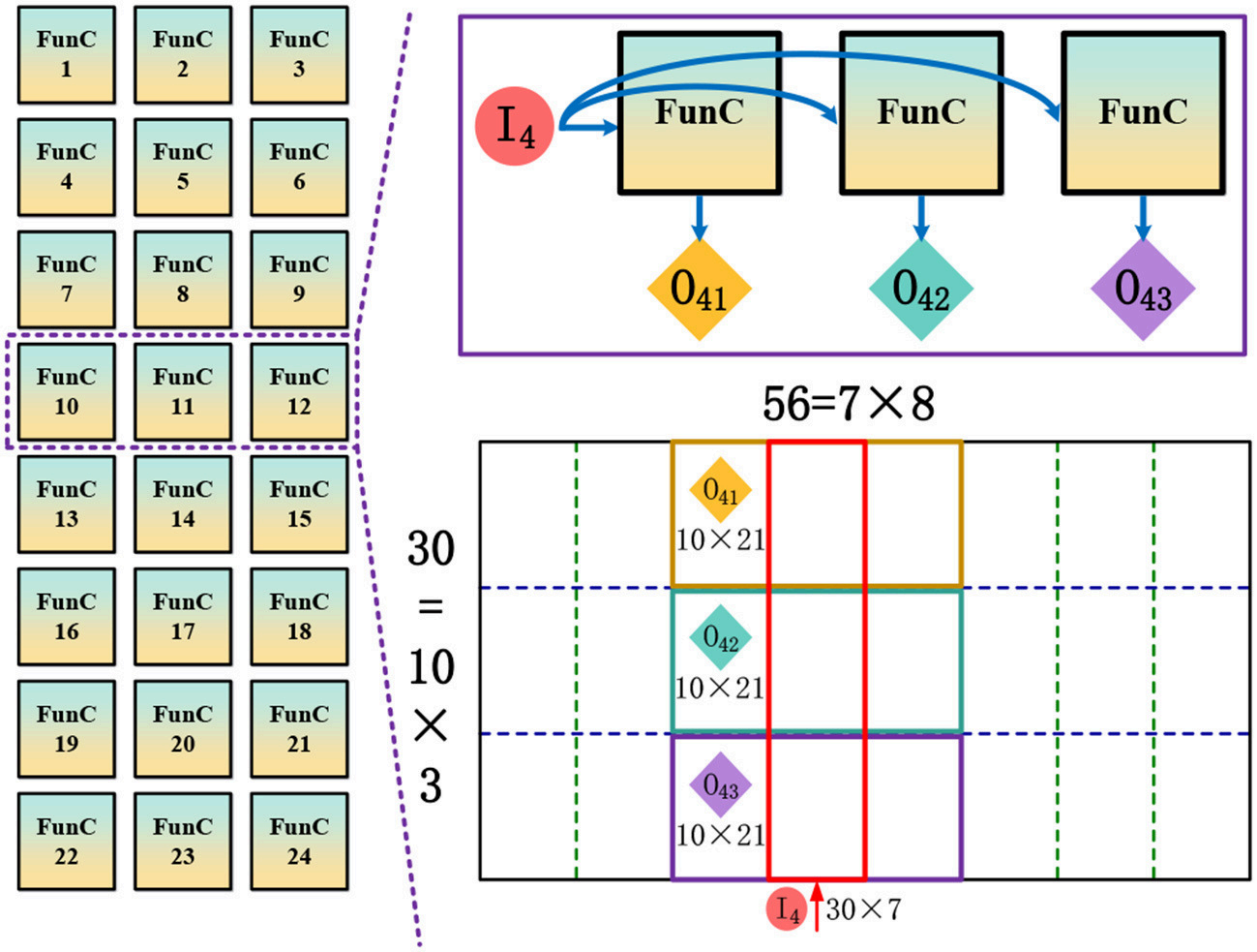
Illustration of the proposed slicing scheme for efficient network mapping.

As shown in Figure 5, first, we partition the 2D locally-connected recurrent network into several column-wise slices, and the slice width is jointly determined by the network height and the fan-in number of each FunC. Here we partition it to 8 slices, wherein each one (such as *I*_4_) contains 30 × 7 ≤ 256 neurons. On the other side, considering that each neuron is only connected to its local CF covering 15 × 15 neurons, each slice is possible to affect the membrane potential of adjacent three slices including itself. For example, *I*_4_ would affect the membrane potential of *I*_3_, *I*_4_ and *I*_5_. However, the total number of output neurons in these affected slices are more than 256. To this end, we further partition the possible outputs of these three column-wise slices to three row-wise slices, e.g., *I*_4_ ⇒ {*O*_41_, *O*_42_, *O*_43_}, wherein each row-wise slice only has 10 × 21 ≤ 256 output neurons. According to the proposed column-wise and row-wise slicing for addressing the issue of limited inputs and outputs, respectively, a minimum block of input neurons, output neurons, and their weighted connections (e.g., *I*_4_ ⇒ *O*_41_, *I*_4_ ⇒ *O*_42_, *I*_4_ ⇒ *O*_43_) could be mapped onto a single FunC. The routing from *I*_4_ to *O*_41_, *O*_42_, and *O*_43_ is handled by a routing strategy different from the regular P2P routing introduced in section 3.2. Here we design an adjacent multicast (AMC) routing in which each FunC can pack its received packets again with a new address of an adjacent FunC (configured in the AMC registers) and send it out. In this way, a source FunC is able to communicate with multiple continuous destination FunCs without increasing the memory cost of routing table. Theoretically, there is no limitation on the number of destination FunCs via this relay-like AMC routing. Compared to the P2P routing, AMC routing is more suitable for the inter-FunC bulky data sharing.

Furthermore, note that the outputs from the FunCs at this step is just a partial potential because each neuron is usually affected by several different column-wise slices. For instance, each neuron in the left part of *O*_41_ is also driven by *I*_2_ and *I*_3_, so its complete state should be obtained by accumulating the corresponding outputs from three adjacent column-wise slices of *I*_2_, *I*_3_ and *I*_4_. This indicates that a second-order accumulation using extra FunCs with VVA operations is required for the complete VMM operation in Step 1. In our implementation, we incorporate this VVA accumulation into Step 2 (to be shown in **Table 5**). Note that here we use a 30 × 56 network with 15 × 15 CF just for case study. The larger size could also be implemented using this slicing scheme at the cost of more resources.

### 4.3. Data quantization

After mapping the network topology, the data quantization becomes an essential step to convert the model in software into its hardware counterpart since the data type and bit width on the NN chip are usually limited. In our NN architecture, all the computations are in the fixed-point format, and the precision for input-weight multiplication and intermediate accumulation is 8 bits and 24 bits, respectively. Actually, the quantization from floating-point data to bit-limited fixed-point data can be transformed to a scaling and rounding problem. For simplification, we use integer and integralization to replace the fixed-point quantization. At each execution step mentioned in section 4.2, we observe a scaling chain rule governed by

(6) $$\{\begin{array}{l} \varphi (x\times y)=z\Leftrightarrow \varphi_{\rho_{F}}(\rho_{x}\cdot x\times \rho_{y}\cdot y)=\frac{\rho_{x}\cdot \rho_{y}}{\rho_{F}}z\Leftrightarrow \rho_{z}=\frac{\rho_{x}\cdot \rho_{y}}{\rho_{F}}\\ \varphi (x+y)=z\Leftrightarrow \varphi_{\rho_{F}}(\rho \cdot x+\rho \cdot y)=\frac{\rho }{\rho_{F}}z\Leftrightarrow \rho_{z}=\frac{\rho }{\rho_{F}} \end{array}$$

where *x* or *y* denotes the original floating-point input or weight at each FunC, *z* is the corresponding output, and ρ_*x*_ (or ρ), ρ_*y*_ (or ρ), and ρ_*z*_ are their scaling coefficients, respectively. Note that an extra bit truncation is required to reduce the bit width of the accumulated potential (24 bits) from dendrite to 10 bits before feeding it into the LUT_Fun, which can reduce the memory cost of LUT. The scaling effect of the bit truncation and LUT can be modeled as an equivalent scaling factor ρ_*F*_, and φ_ρ_*F*__ denotes both the truncation and LUT_Fun. If the LUT function is a linear function (or piecewise linear function, such as *ReLU*), Equation (6) is valid for describing a linear scaling relationship, termed as a linear scaling chain rule in this paper. This chain rule indicates the scaling factor of neuronal output at the *l*-th execution step can be deterministically derived based on the output value at the (*l*-1)-th step, and can be propagated step by step. Equation (6) only describes the scaling relationship, and doesn't include the rounding operation. Updating the equation to include rounding, it becomes

(7) $$\{\begin{array}{l} \varphi (x\times y)=z\Leftrightarrow \phi (\varphi_{\rho_{F}}(\phi (\rho_{x}\cdot x)\times \phi (\rho_{y}\cdot y)))\approx \frac{\rho_{x}\cdot \rho_{y}}{\rho_{F}}z\Leftrightarrow \rho_{z}\approx \frac{\rho_{x}\cdot \rho_{y}}{\rho_{F}}\\ \varphi (x+y)=z\Leftrightarrow \phi (\varphi_{\rho_{F}}(\phi (\rho \cdot x)+\phi (\rho \cdot y)))\approx \frac{\rho }{\rho_{F}}z\Leftrightarrow \rho_{z}\approx \frac{\rho }{\rho_{F}} \end{array}.$$

where ϕ(·) is the rounding operation. Equation (7) is equivalent to adding random noise to the original chain rule shown in Equation (6).

**Figure 6:**
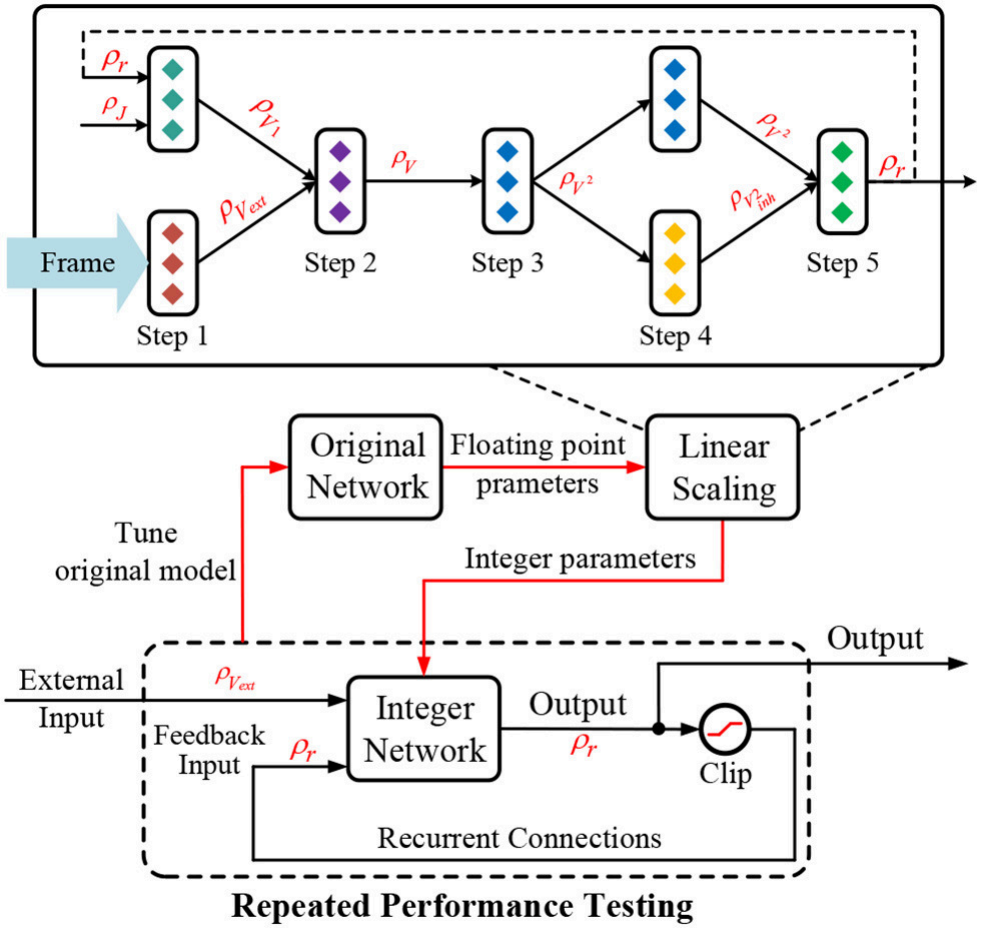
Quantization with a linear scaling chain rule in the feedforward pass corresponding to the five execution steps mentioned in section 4.2 and an extra constant-scaling restriction in the feedback pass.

The proposed linear scaling chain rule can describe the scaling effect well as data propagates in a feedforward structure under the quantization constraint. However, the recurrent network has a feedback connection that will influence the normal data scaling. First, as shown in Figure 6, each difference iteration in the forward pass subjects to the above linear scaling chain rule across the five execution steps. Second, the firing rate at time phase *t* will be fetched back to the network as the input for next phase *t* + 1. So the overall scaling factor on firing rate at each iteration should keep unchanged, i.e., a constant ρ_*r*_, otherwise the firing rate will become larger and larger or smaller and smaller causing state explosion or vanishing issue, respectively. To this end, we have to configure the scaling factor of connection weights (ρ_*J*_) and truncation/LUT_Fun (ρ_*F*_) in each FunC to guarantee a constant-scaling restriction on the input/output firing rate after the feedforward scaling propagation. This is a typical closed-loop control that requires repeated verification, i.e., testing the network performance and adjusting the hardware configuration or modifying the original floating-point parameters until a satisfactory result is achieved. It is worthy noting that the rounding operations would introduce random errors, but the simulation results show that the CANN model can tolerate noises to a great extent, which was also mentioned in Martel and Sandamirskaya (2016). To avoid possible data overflow caused by the rounding noise, we enforce a clipping operation to keep the data in limited range, such as [-128, 127] under 8-bit quantization.

## 5. Experimental results

### 5.1. Experimental setup

**Figure 7:**
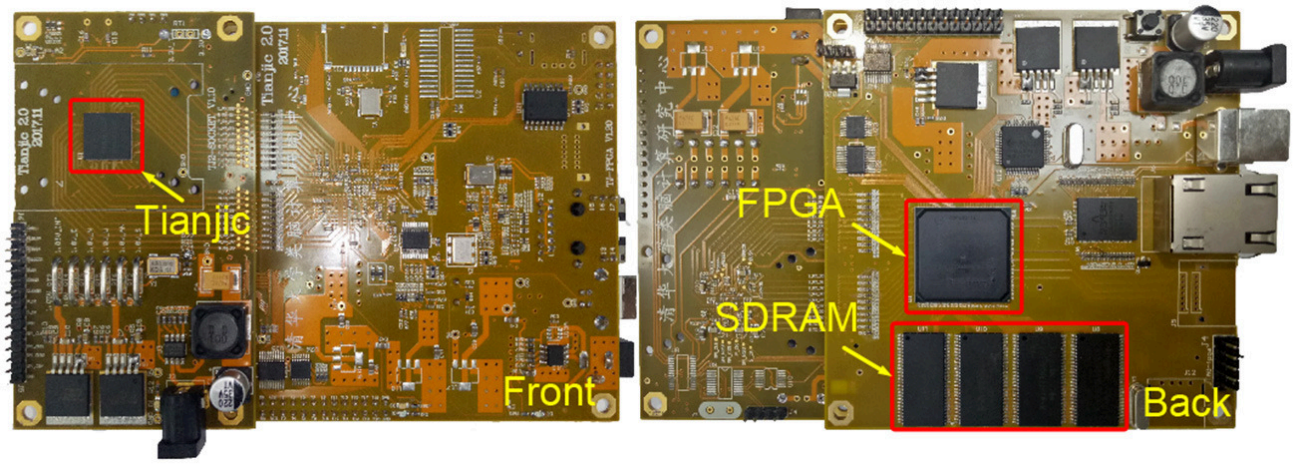
Single-chip PCB.

The simulation environment for the algorithm analysis is based on a PC with Intel i7 6700K CPU (4GHz) and Matlab R2017a software. For the hardware validation, we fabricate a chip in UMC 28nm HLP CMOS process (named Tianjic) to implement the many-core NN architecture described in section 3 along with the AMC routing strategy mentioned in section 4.2. To emulate the object tracking scenario, we develop a single-chip PCB equipped with an Altera Cyclone 4 FPGA and four SDRAMs (total 128 MB), as shown in Figure 7. Tianjic accommodates the tracking model with pre-programmed synaptic weights. The resized video is pre-stored in SDRAM and then injected into the NN chip through FPGA. Table 2 lists the chip configuration. Considering the fabrication cost, we only integrate 156 FunCs onto one chip. With 300 MHz clock, the chip can finish all computations and communications in 16.8 μ*s* during each time phase which reflects the minimum phase latency for guaranteeing the running correctness. The power consumed by each FunC is 1.95~6.29 mW in different operation modes (Table 1) or idle mode, which includes the chip-level overhead. Other components on PCB consume 5.5 W in total. Although only a single chip is enough in this work, we also design an inter-chip communication infrastructure for supporting multi-chip scalability if larger networks are required, which is compatible with the intra-chip routing strategies (P2P and AMC). Specifically, four bidirectional LVDS (low voltage differential signaling) interfaces are incorporated at each of the four chip sides.

**Table 2:** Chip configuration.

**FunCs per chip**	**MACs per FunC**	**Synapse array per FunC**
12 × 13	16	256 × 256 (SRAM)
**Data precision**	**Clock frequency**	**Phase latency**
8 bits (I/O)	300 MHz	16.8 μs

**Figure 8:**
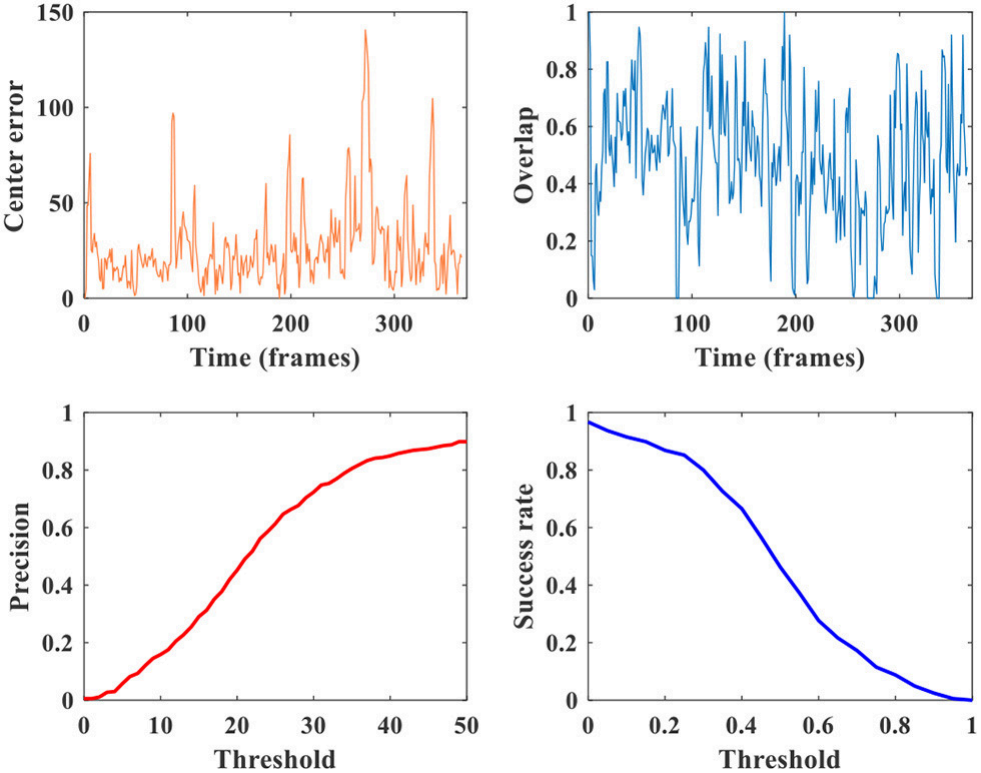
Comparison of several metrics: center error, precision, overlap, and success rate.

**Figure 9:**
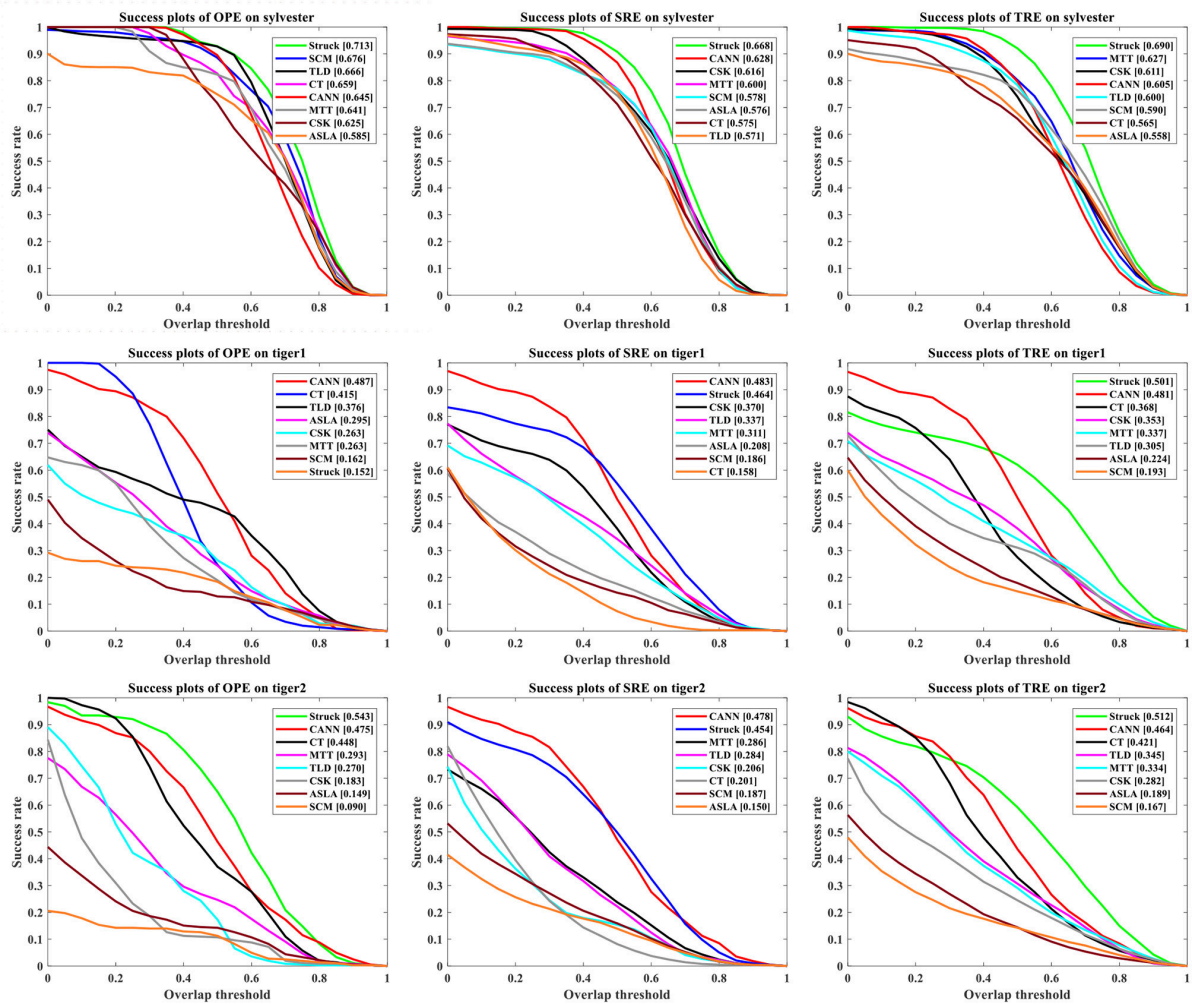
Success rate of OPE, SRE and TRE, wherein the overall AUC score is listed in the legend. For clarity, only 8 trackers on three videos are plotted.

**Figure 10:**
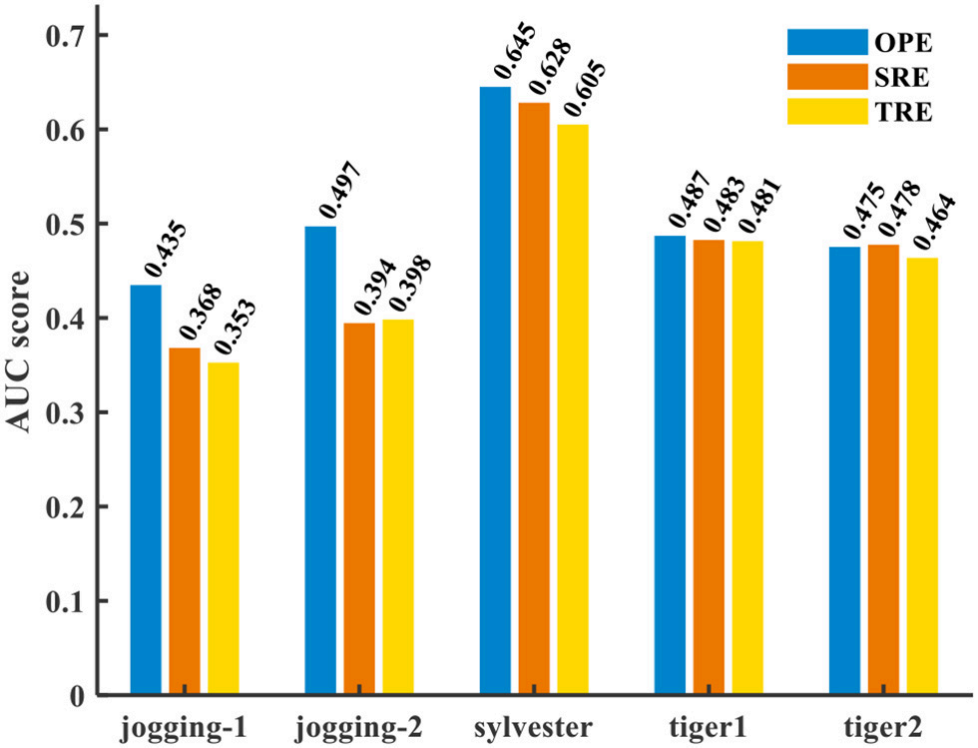
AUC score of CANN on several videos.

**Figure 11:**
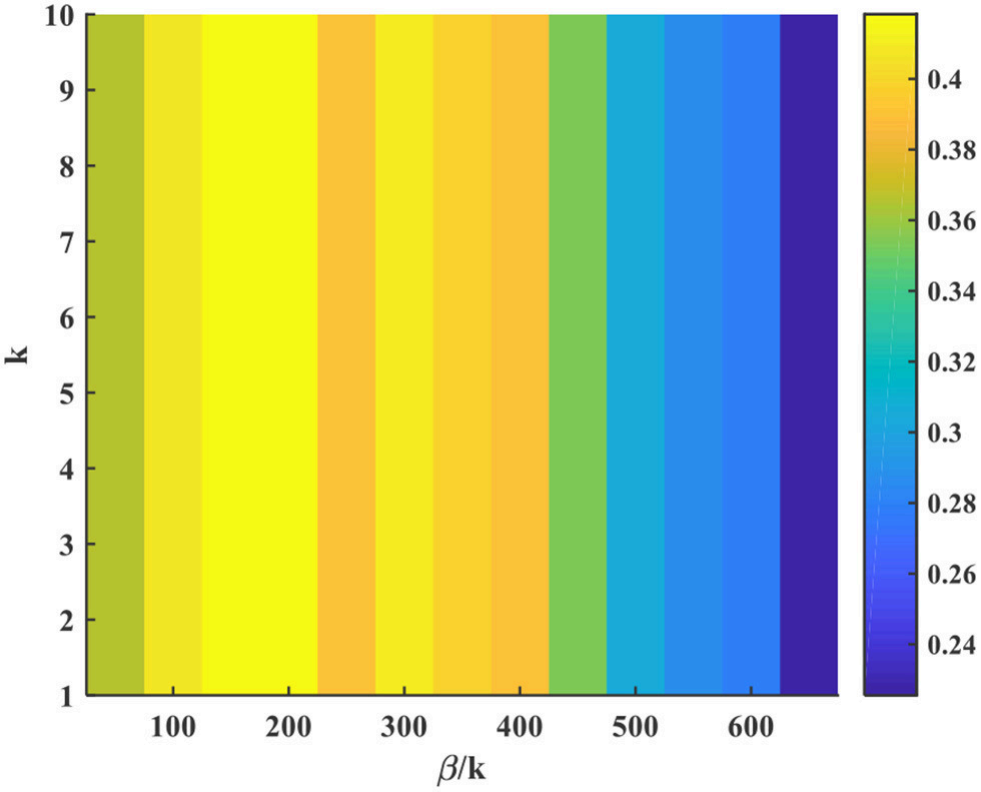
Influence of the model hyper-parameters.

**Figure 12:**
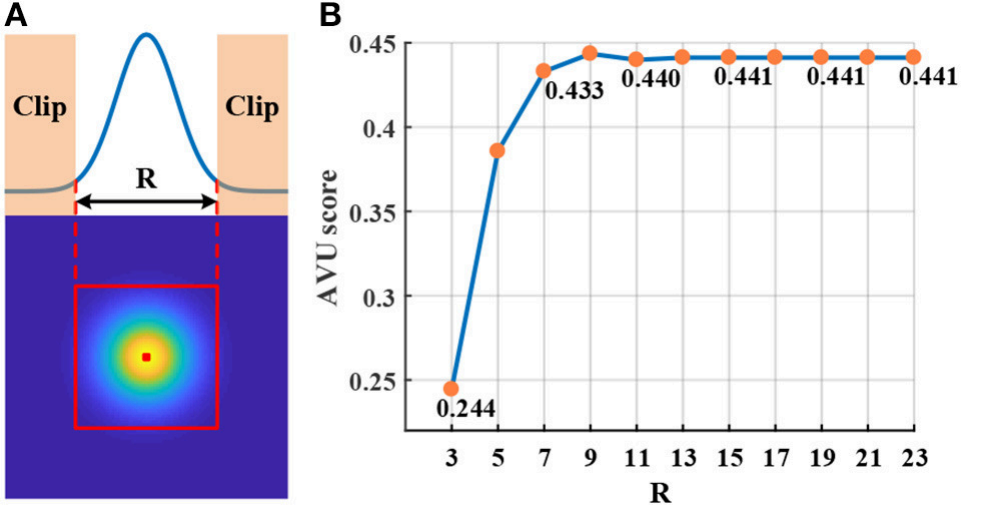
Influence of the connection area: **(A)** CF clipping; **(B)** AUC score as R size increases.

**Figure 13:**
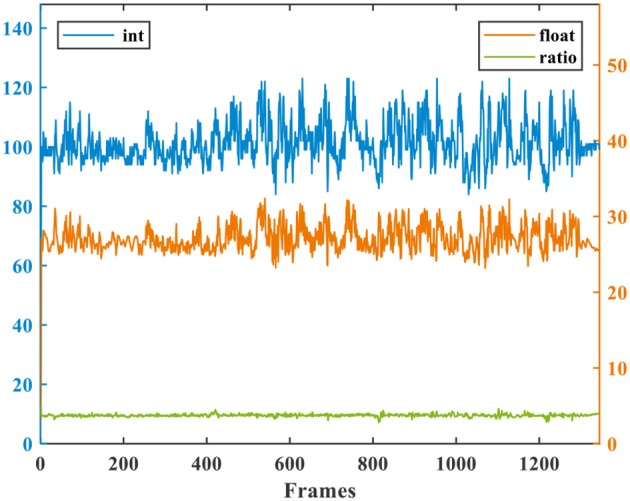
The constant-scaling restriction for the data quantization of recurrent networks.

**Figure 14:**
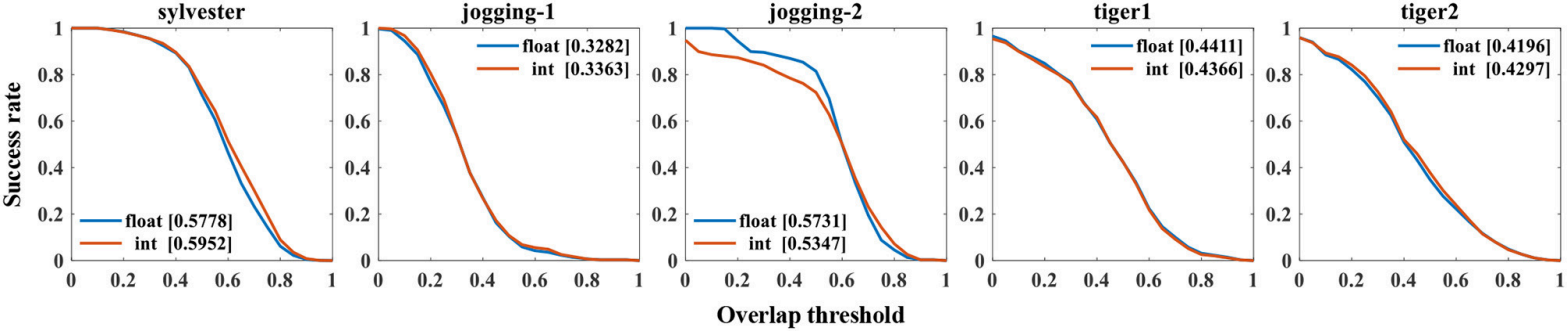
Influence of the data quantization.

**Figure 15:**
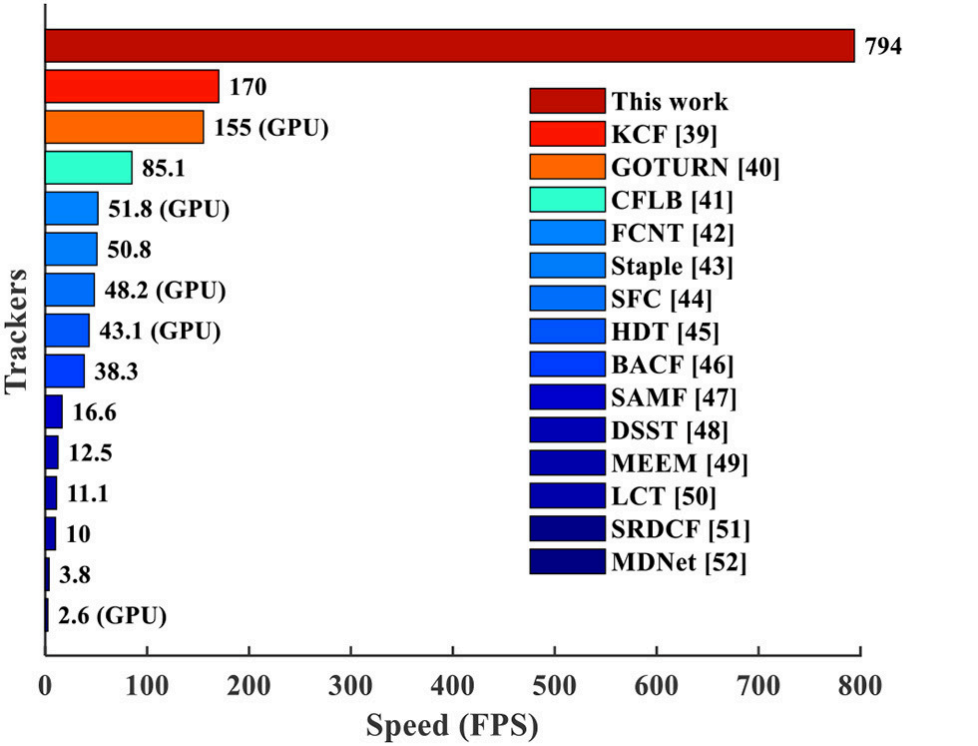
Speed comparison with existing tracking algorithms (Danelljan et al., 2014a,b, 2015; Zhang et al., 2014; Galoogahi et al., 2015, 2017b; Henriques et al., 2015; Ma et al., 2015; Wang L. et al., 2015; Bertinetto et al., 2016a,b; Nam and Han, 2016; Qi et al., 2016). The data marked with “(GPU)” are tested on GPU while others are on CPU.

**Figure 16:**
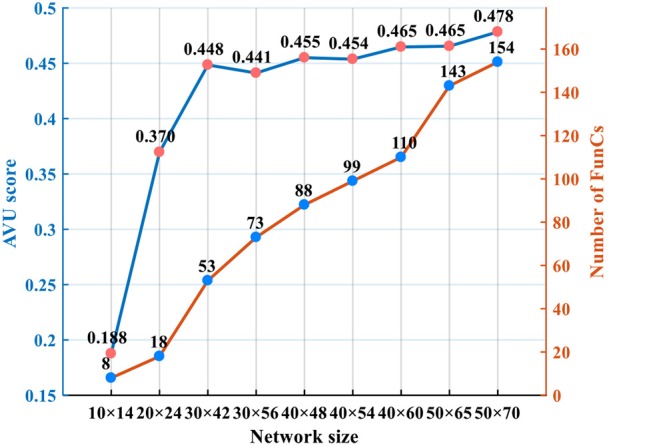
Trade-off between tracking accuracy and resource overhead as the network size increases.

**Figure 17:**
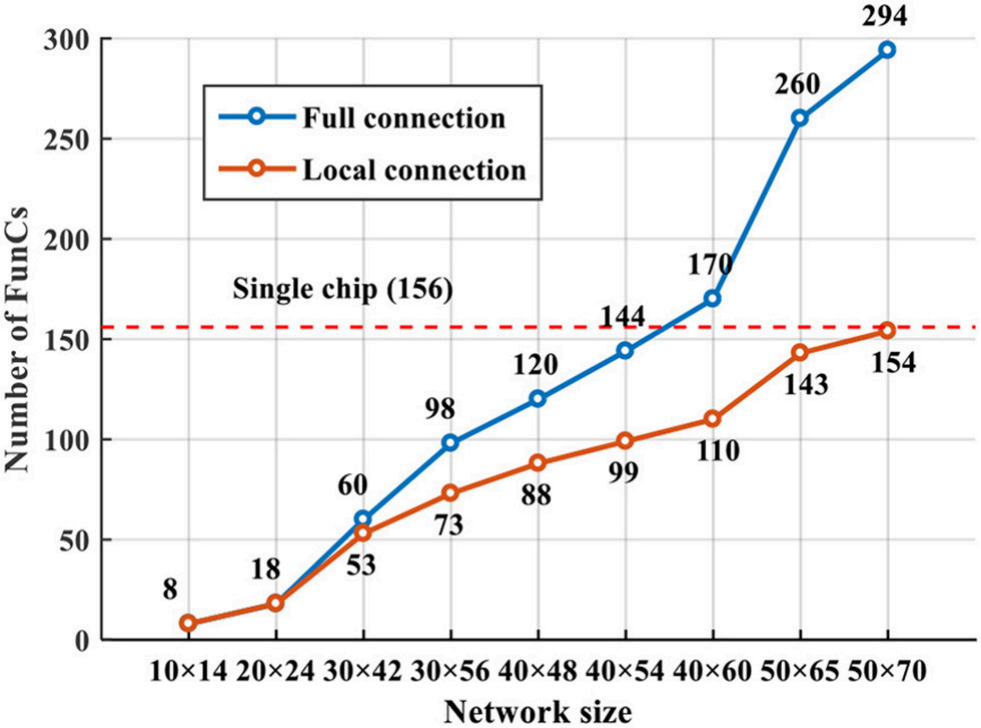
Resource overhead before and after adding the local-connection restriction.

We test the CANN tracking on several video datasets from OTB-13 (Wu et al., 2013) and OTB-15 (Wu et al., 2015), the video attributes of which are shown in Table 3. For each difference frame, we execute 15 iterations of Equation (5). Regarding the experimental evaluation, the accuracy results are simulated in Matlab (Figures 8–**11**, **16** don't consider any hardware constraints while **Figures 12–14** incorporate the hardware constraints on connection and data bit width), and the resource overhead and tracking speed (involving Figures **15**–**17** and **Table 5**) come from chip simulator and real measurements.

**Table 3:** Information about the five video workloads.

**Video**	**Frames**	**Attributes**
Jogging-1	307	OCC, DEF, OPR
Jogging-2	307	OCC, DEF, OPR
Sylvester	1345	IV, IPR, OPR
Tiger1	354	IV, OCC, DEF, MB, FM, IPR, OPR
Tiger2	365	IV, OCC, DEF, MB, FM, IPR, OPR, OV

*IV, Illumination Variation; OCC, Occlusion; DEF, Deformation; MB, Motion Blur; FM, Fast Motion; IPR, In-Plane Rotation; OPR, Out-of-Plane Rotation; OV, Out-of-View. More details can be found in Wu et al. (2013)*.

### 5.2. Model analysis

#### 5.2.1. Evaluation metrics

Several metrics are widely used for quantitative evaluation of tracking accuracy (Cehovin et al., 2016). At this stage, none of them is a killer standard. To compare these different methods, we first recall some general definitions. An object location set in a frame sequence with length *T* is defined as

(8) $$\Lambda ={\left\{\left(R_{t},{\text{x}}_{t}\right)\right\}}_{t=1}^{T}$$

where *R*_*t*_ denotes the cover region of the bounding box and x_*t*_ is the center location of the object.

The center error measures the difference between the predicted and ground-truth center, which is defined as the average Euclidean distance in pixel units. Denote ${\text{x}}_{t}^{P}$ as the predicted center location and ${\text{x}}_{t}^{G}$ as the ground-truth one. Then the center error over all frames in one sequence is governed by

(9) $$\Delta (\Lambda^{P},\Lambda^{G})=\frac{1}{T}\sum_{t=1}^{T}\delta_{t},\;\;\;\delta_{t}=\|x_{t}^{P}-x_{t}^{G}\|.$$

Center error is used to evaluate the overall tracking accuracy for a sequence. Usually, the precision further describes the percentage of accurately predicted centers (within a given distance threshold).

Another evaluation metric is the overlap, which is determined by the intersection area between the predicted and ground-truth bounding boxes. This measure accounts for both the location and size of the object, and does not result in extremely large errors at tracking failures. Given the predicted bounding box $R_{t}^{P}$ and the ground-truth bounding box $R_{t}^{G}$, the overlap of one sequence is defined as

(10) $$\Phi (\Lambda^{P},\Lambda^{G})={\left\{\phi_{t}\right\}}_{t=1}^{T},\;\;\;\;\phi_{t}=\frac{\left|R_{t}^{P}\cap R_{t}^{G}\right|}{\left|R_{t}^{P}\cup R_{t}^{G}\right|}$$

where ∩ is the intersection, ∪ is the union, and |·| denotes the number of pixels in the corresponding region. Furthermore, the success plot records a curve wherein each point represents the percentage of the accurately predicted bounding boxes (with overlap larger than a given threshold). The overall success score is defined as the area under curve (AUC). It can be proved that the AUC equals to average overlap (Cehovin et al., 2016).

For an intuitive understanding of these metrics, Figure 8 presents a comprehensive visualization of the tracking accuracy under different metrics on video tiger2. The center error and overlap are curves having nothing to do with the threshold, and they usually fluctuate along the temporal dimension (i.e., frame). While every point on the precision or success plot corresponds to an overall accuracy obtained from the center errors or overlaps across all frames under a pre-given comparison threshold, respectively. Note that the precision, overlap and success rate are all in [0, 1]. Because the success rate and AUC score provide number within [0, 1] (including both accuracy and threshold) and does not fluctuate, we mainly use them for evaluating our model in the following sections.

#### 5.2.2. Tracking accuracy

To analyze the tracking accuracy comprehensively, we adopt the tests of one-pass evaluation (OPE), temporal robustness evaluation (TRE), and spatial robustness evaluation (SRE). Specifically, OPE is to run trackers throughout the whole sequence using ground truth of the first frame as initialization. This is a simple but useful way to evaluate trackers. For the robustness evaluation, TRE and SRE can be applied. In TRE, the whole sequence is split into several segments, then the influence of initialization location can be analyzed (the first frame of each segment can calibrate the initialization). SRE is to sample the initial bounding box in the first frame by shifting or scaling the ground truth, which focuses on the spatial robustness. Please refer to Wu et al. (2013) for more detailed information. Table 4 provides our parameter configuration for all the model analysis experiments. The reason why we only give the β/*k* value rather than individual β and *k* will be explained latter.

**Table 4:** Parameter configuration.

**Video**	**β/*k***	**Network size**	**CF size**
Sylvester	300		
Jogging-1	600		
Jogging-2	900	96 × 128	15 × 15
Tiger1	200		
Tiger2	100		

Figure 9 presents the success rate compared to existing trackers under OPE, TRE, and SRE tests. For figure clarity, only 8 trackers on three videos (sylvester, tiger1, tiger2) are shown. We can see that the CANN model performs quite well, which can approach or surpass other trackers. The overall success scores on all five videos are further shown in Figure 10. CANN presents advanced success scores across all these videos. Recalling Table 3, the sylvester video is in a simpler environment (e.g., with less occlusion, deformation, and motion blur), so all trackers (including CANN) perform the best on it.

Figure 11 analyzes the influence of the model hyper-parameters, *k* and β in Equation (5). Here we test on video tiger2. The X axis represents the ratio between β and *k*, i.e., β/*k*, and the Y axis is *k*. The color indicates different AUC score. We can see that the individual value of *k* (also reflecting the β value under the same β/*k* condition) has little impact on the tracking accuracy. In contrast, the ratio β/*k* heavily affects the AUC score. The underlying mechanism lies in Equation (5), in which we can find that the state of membrane potential *V*(**x**, *t*) is only determined by the ratio of β/*k* if we substitute the firing rate *r*(**x**, *t*) into the difference equation of *V*(**x**, *t*). When β/*k* is fixed, *V*(**x**, *t*) keeps unchanged under the same external stimulus. In this case, the bump pattern of firing rate remains the same or with an overall scaling effect under different *k* value, which will not affect the object prediction because it is only determined by the location of central neuron with the maximum firing rate.

### 5.3. System analysis

#### 5.3.1. Influence of hardware constraints

Mapping the original CANN model onto the many-core NN hardware, two major constraints must be considered: connection and precision. The former one is caused by the limited wiring resources resulting in limited fan-in and fan-out connectors on a single FunC. This restricts the connection number of each neuron and the overall network size as well. The latter one is caused by the limited compute and memory resources which makes it impractical for high-precision floating-point operations. In our design, we just take 8 bits as a case study, but it is easy to extend to other precisions.

First, Figure 12 shows the influence of CF size (i.e., *R*) in the local-connection restriction (on video tiger1). We can see that larger *R* usually generates higher AUC score. However, the AUC score gradually saturates when *R* is sufficiently large. From the guidance of this result, we configure the CF size to be 15 × 15 in our experiments to achieve both the best accuracy and fewest connections.

Then, we investigate the precision influence. By using our quantization method proposed in section 4.3, the overall firing rate curve is equivalent to scale the original floating-point curve by a constant factor of ρ_*r*_, which is shown in Figure 13 (on video sylvester). The constant-scaling restriction for recurrent networks is critical to address the state explosion or vanishing issue. The slight fluctuation of the scaling ratio is caused by the aforementioned rounding noise in Equation (7). Figure 14 further shows the comparison of tracking accuracy before and after data quantization on all videos. The AUC scores are shown in the legend for each sub-figure. We can see that the “feedforward linear scaling chain rule & feedback constant scaling” quantization method proposed in Figure 6 is effective and causes little accuracy loss. The object in jogging-2 video has the similar color with the backgrounds, so the tracking accuracy presents a slightly larger degradation.

#### 5.3.2. System performance

Here we take a CANN model with 30 × 56 network size as an example to show the hardware implementation. Table 5 illustrates the mapping details and the resource overhead in all the execution steps mentioned in section 4.2. Step 1 for the integration of recurrent inputs consumes the most resources due to the heavy VMM operation. By leveraging the slicing scheme proposed in section 4.2, it generates the partial membrane potential using 24 FunCs as shown in Figure 5. Each 3 FunCs for one column-wise slice share the same inputs through AMC routing. The following steps only involve vector operations, such as VVA, VVM, VB, and VS. In these operations, the output data from previous step is dynamically buffered in synapse, different from the static weights in VMM operation at Step 1. To provide enough inputs for the next step, the outputs from Step 2, Step 3, and Step 4-1 have to be copied through configuring FunCs that have the same parameters and modes and share packets through AMC routing. On the contrary, the inter-step communication still uses P2P routing, since the bulky AMC routing is invalid in these cases for routing to the next step due to the requirements for different input data or addresses in post-FunCs. In Step 4-1, the nonlinear LUT should be configured to calculate the division for producing the inhibition factor. The potential delay is used for timing alignment that guarantees the correct dataflow step by step. Totally, 73 FunCs are enough for this CANN model, which indicates that we can finish object tracking task on one single chip with 156 FunCs shown in Figure 7 and Table 2.

**Table 5:** Mapping details and resource overhead at every step for a 30 × 56 CANN example.

**Step**	**Functionality**	**Implementation**	**Operation**	**No. FunC**
		8 column-wise slices		
1	Recurrent input	3 FunCs for each column-wise slice	VMM	3 × 8
		30 × 7 fan-ins and 10 × 21 fan-outs for each FunC		
2	Membrane potential	Integrate 3 partial potentials and external stimulus	VVA	7 × 2
	(2 copies)	240 × 4 fan-ins and 240 fan-outs for each FunC		
3	Potential squared	240 × 2 fan-ins and 240 fan-outs for each FunC	VVM	7 × 2
	(2 copies)			
4-1	Inhibition factor	30 × 56 fan-ins and 1 fan-out for each FunC	VVA + Lat_Acc	1 × 7
	(7 copies)		nonlinear LUT_Fun	
4-2	Potential delay	240 fan-ins and 240 fan-outs for each FunC	VB	7
5	Firing rate	241 fan-ins and 240 fan-outs for each FunC	VS	7
				Total: 73

Figure 15 compares the throughput of the CANN object tracking on our many-core chip with those on conventional CPU and GPU. By using the modified CANN model and our mapping framework, the many-core NN architecture holds great potential for fast object tracking. Specifically, we implement the CANN model with 5 execution steps and one time phase for each step. According to the chip performance of 16.8μ*s* phase latency (Table 2) and 15 iterations for each difference frame, a throughput of 794 frames-per-second (FPS) can be achieved. This is significantly faster than the advanced trackers (4.7x-305x) on CPU or GPU. Note that the throughput in Figure 15 means the speed based on pre-stored resized video, which doesn't count the time for video resizing itself. In this paper, we focus on the efficient execution of tracking model rather than the video pre-processing which can be completed by anterior camera circuits. In fact, previous work (Carey et al., 2012) reported ultra-fast speed 100,000 FPS for closed-shape detection on vision chip with analog-digital mixed signals. However, the application scenario is very different, so we don't include it into our comparison.

Regarding the scalability of network size, it is a software-hardware trade-off. As shown in Figure 16 (on video tiger1), it is possible to achieve better tracking accuracy if we deploy larger network (remaining *R* = 15). However, larger network causes exponentially increasing resource consumption. In real-world applications, it is better to determine the network size according to the requirement for tracking accuracy. From Figure 16 we can see that, with 156 FunCs per chip, we still have space for scale increasing (e.g., up to 50 × 70 network size). Smaller than this threshold, one single chip is enough; otherwise, we should consider multi-chip interconnection. In fact, our many-core architecture is fully scalable, as shown in Figure 3B, which makes it easy to be extended to a multiple-chip system mentioned in section 5.1. The similar 2D mesh network-on-chip with the inter-FunC/intra-chip communication is also compatible with the inter-chip communication. The only difference is that a merge-split technique is needed for the inter-chip interface due to the limited chip I/O.

Figure 17 shows the comparison of resource overhead before and after adding the local-connection restriction proposed in Section 2. For small networks, the resource saving is not significant since each input slice with CF size of 15 × 15 probably has impacts on the states of all slices, which degrades to the fully-connected case. For larger networks, the local-connection restriction gradually helps reduce the resources since each input slice only affects its neighboring slices. With this adaption, a single chip can accommodate a network of up to 50 × 70 size (consuming 154 FunCs); while without it, a smaller network (e.g., 40 × 60 size consuming 170 FunCs) already exceeds the resources of one single chip.

## 6. Conclusion and discussion

In this paper, we adapt and map the CANN model onto the many-core NN architecture for fast object tracking. By adding a restriction for distance-aware local connection, we remove most remote connections to make the model hardware-friendly. Then we design a many-core NN architecture with five vector/matrix operations in dendrite and three transformation operations in soma to cover all the computations in the CANN model. A mapping framework is further built for deploying the model onto the NN hardware, which includes three stages: dynamics discretization, topology mapping, and data quantization. Based on the five discrete execution steps, a slicing scheme for efficient topology mapping and a constant-restricted scaling chain rule for lossless data quantization are elaborated. Comprehensive tracking analysis is demonstrated and a real chip is fabricated for performance evaluation. Putting the tracking model onto one single chip, we achieve comparable tracking accuracy and fast tracking speed (nearly 800 FPS). This work enables high speed for tracking applications in scenarios with limited resources and energy, such as in embedded systems.

Besides the emphasized compact end-to-end model and fast tracking speed, next we discuss more on the advantage-disadvantage trade-off of the proposed solution. First, the continuous dynamics makes the CANN model suitable for continuous tracking with smooth trajectory. However, it is still challenging for it to tackle well in the complex scenarios with strong disturbance from other close objects or variable backgrounds. In those cases, the CANN model performs worse than the detection-recognition combined methods (Wang and Yeung, 2013; Hong et al., 2015). Second, CANN is driven by difference signal that makes it more sensitive to the object edge rather than the center. This will add noise on the recognized bounding box and degrade the tracking accuracy based on current evaluation metrics. In any case, our framework indeed provides an efficient solution for fast tracking on the widely used many-core NN architecture. In the scenarios with simpler environment but strong dependency on high speed, this solution presents a great potential. The single-chip accommodation also makes it suitable for various embedded systems with constraints on resources and power.

## Author contributions

LD and ZZ proposed the idea, designed and did the experiments. LD, ZZ, LingL, and XH conducted the modeling work. LD, XM, GW, LiuL and JP conducted the design and testing of the tracking system. LD, ZZ and GL wrote the manuscript, then YX revised it. GL, JP, and YX directed the project and provided overall guidance.

### Conflict of interest statement

The authors declare that the research was conducted in the absence of any commercial or financial relationships that could be construed as a potential conflict of interest.
